# Complete Genome Sequence of *Riemerella anatipestifer* Serotype 10 Strain HXb2

**DOI:** 10.1128/genomeA.00278-17

**Published:** 2017-05-04

**Authors:** Qinghai Hu, Jingjing Qi, Huimin Bo, Guangqing Liu, Minjie Tao, Yunzhu Ding, Yafei Xue

**Affiliations:** Shanghai Veterinary Research Institute, Chinese Academy of Agricultural Sciences, Shanghai, China

## Abstract

The complete genome sequence of highly virulent *Riemerella anatipestifer* strain HXb2 was determined. The genome consisted of a single circular chromosome of 2,425,237 bp containing 2,383 putative open reading frames (ORFs), 9 rRNA operons, and 40 tRNA genes.

## GENOME ANNOUNCEMENT

*Riemerella anatipestifer*, a Gram-negative bacterium belonging to the family *Flavobacteriaceae*, is the etiological agent of a contagious septicemic disease in ducks, geese, turkeys, and other birds, which causes serious economic losses to the duck industry worldwide due to high mortality, weight loss, and condemnations (1). Twenty-one serotypes of *R. anatipestifer* with little or no cross-protection among them have been identified thus far. Serotypes 1, 2, 10, and 6 have been responsible for most of the major outbreaks in China (2, 3). There are several genomic resources available for *R. anatipestifer* (4–7). *R. anatipestifer* serotype 10 strain HXb2, which was isolated from a sick duck in China by Q. Hu 15 years ago, is a highly virulent strain with a median lethal dose of 82 CFU to 8-day-old Cherry Valley ducklings (8). Furthermore, the gene coding for siderophore-interacting protein, which was found in most of the detected *R. anatipestifer* strains and involved in iron acquisition and virulence of *R. anatipestifer*, was not found in strain HXb2 (9), indicating that it may have an iron utilization system unique from those of other *R. anatipestifer* strains. The genome sequence may provide essential information to further clarify these phenomena. Therefore, the complete sequence of this strain was sequenced using the PacBio RSII long-read sequencing method. A 10-kb genomic DNA library was constructed using the PacBio SMRTbell template prep kit (PacBio, Menlo Park, CA, USA) and sequenced with a PacBio RS II sequencer (PacBio). Then, to fill the gaps between contigs, an additional 300-bp genomic DNA library was constructed using the NEBNext UltraTM DNA library prep kit (New England BioLabs, Ipswich, MA, USA) and sequenced with a HiSeq^TM^ 2500 sequencer (Illumina, Inc., San Diego, CA, USA). The sequencing information was assembled with RS_HGAP_assembly.3, with a genome coverage of 49 folds. Putative coding sequences (CDSs) were identified with Grammer version 3 (10). The tRNA and rRNA genes were predicted using tRNAscan-SE and RNAmmer, respectively (11, 12). Functional annotations of CDSs were performed by searching against the nonredundant protein database using the BLASTP algorithm (13). Genomic islands were predicted with IslandViewer 3.

The genome of strain HXb2 consists of a circular chromosome of 2,425,237 bp with a G+C content of 34.97% and 2,383 total putative open reading frames (ORFs) with nine rRNA operons and 40 tRNA genes. In addition, nine genomic islands were predicted in the genome sequence of strain HXb2.

### Accession number(s).

The complete genome sequence of strain HXb2 has been deposited in GenBank under the accession number CP011859.
